# A Pilot Study Investigating Changes in the Human Plasma and Urine NAD+ Metabolome During a 6 Hour Intravenous Infusion of NAD+

**DOI:** 10.3389/fnagi.2019.00257

**Published:** 2019-09-12

**Authors:** Ross Grant, Jade Berg, Richard Mestayer, Nady Braidy, James Bennett, Susan Broom, James Watson

**Affiliations:** ^1^Australasian Research Institute, Sydney Adventist Hospital, Wahroonga, NSW, Australia; ^2^Sydney Adventist Hospital Clinical School, University of Sydney, Sydney, NSW, Australia; ^3^School of Medical Sciences, Faculty of Medicine, University of New South Wales, Sydney, NSW, Australia; ^4^NAD+ Research Inc., Springfield, LA, United States; ^5^Springfield Wellness Center, Springfield, LA, United States; ^6^School of Psychiatry, University of New South Wales, NPI, Euroa Centre, Randwick, NSW, Australia; ^7^School of Natural and Behavioural Sciences, William Carey University, Hattiesburg, MS, United States; ^8^Division of Plastic Surgery, Clinical Faculty, David Geffen School of Medicine, University of California, Los Angeles, Los Angeles, CA, United States

**Keywords:** NAD+, nicotinamide (NAM), ADP ribose, methyl nicotinamide, nicotinamide mononucleotide

## Abstract

Accumulating evidence suggests that active maintenance of optimal levels of the essential pyridine nucleotide, nicotinamide adenine dinucleotide (NAD+) is beneficial in conditions of either increased NAD+ turnover or inadequate synthesis, including Alzheimer’s disease and other neurodegenerative disorders and the aging process. While studies have documented the efficacy of some NAD+ precursors such as nicotinamide riboside (NR) in raising plasma NAD+, no data are currently available on the fate of directly infused NAD+ in a human cohort. This study, therefore, documented changes in plasma and urine levels of NAD+ and its metabolites during and after a 6 h 3 μmol/min NAD+ intravenous (IV) infusion. Surprisingly, no change in plasma (NAD+) or metabolites [nicotinamide, methylnicotinamide, adenosine phosphoribose ribose (ADPR) and nicotinamide mononucleotide (NMN)] were observed until after 2 h. Increased urinary excretion of methylnicotinamide and NAD+ were detected at 6 h, however, no significant rise in urinary nicotinamide was observed. This study revealed for the first time that: (i) at an infusion rate of 3 μmol/min NAD+ is rapidly and completely removed from the plasma for at least the first 2 h; (ii) the profile of metabolites is consistent with NAD+ glycohydrolase and NAD+ pyrophosphatase activity; and (iii) urinary excretion products arising from an NAD+ infusion include NAD+ itself and methyl nicotinamide (meNAM) but not NAM.

## Introduction

The parent pyridine nucleotide, nicotinamide adenine dinucleotide (NAD+) is present in all cells of the body and is essential for cell viability and function.

As a cofactor responsible for electron transport into the respiratory chain, NAD+ and its redox couple NADH are central to energy (ATP) production in the mitochondria *via* oxidative phosphorylation. The phosphorylated metabolite of NAD+, NADP+ with its redox couple NADPH also provides the reducing power to drive a number of anabolic reactions, including cholesterol and nucleic acid synthesis, elongation of fatty acids and regeneration of glutathione (GSH), one of the body’s main antioxidants. Overall this family of pyridine nucleotides, [NAD(P)(H)], contributes to the redox exchange of over 400 enzyme reactions. Importantly, when acting as a redox couple NAD+ is not consumed. However, NAD+ also serves as a substrate for a number of other important metabolic processes and is therefore consumed as a consequence of their chemical reactions, potentially depleting the tissue of NAD+. Included in this number are reactions driven by the poly adenosine phosphoribose-ribose (ADPR) family of enzymes (PARP 1–17) controlling DNA repair and nuclear stability, epigenetic control enzymes (Sirt1–7), intercellular immune communication (CD38/CD157) and neuronal regeneration (SARM1; Essuman et al., 2017). The potential exists therefore for the disruption of cellular metabolism at multiple levels in conditions where NAD+ consumption by these enzymes exceeds NAD+ supply or synthesis.

The clinical importance of maintaining cellular NAD^+^ levels was established early in the last century with the finding that pellagra, a disease characterized by diarrhea, dermatitis, dementia and death, could be cured with foods containing the NAD^+^ precursor niacin (also known as vitamin B_3_; Goldberger, 1914). Although pellagra is rare in developed countries, cellular concentrations of NAD^+^ have been shown to decrease under conditions of increased oxidative damage such as occur during aging (Braidy et al., 2011; Massudi et al., 2012; Guest et al., 2014). Altered levels of NAD^+^ have been found to accompany several disorders associated with increased oxidative/free radical damage including diabetes (Wu et al., 2016), heart disease (Pillai et al., 2005), age-related vascular dysfunction (Csiszar et al., 2019), ischemic brain injury (Ying and Xiong, 2010), misfolded neuronal proteins (Zhou et al., 2015) and Alzheimer’s dementia (Abeti and Duchen, 2012).

In addition to the pathological hallmarks of Aβ plaques and neurofibrillary tau tangles, oxidative damage is a consistent finding in Alzheimer’s disease and is widely recognized as an early event in the pathogenic process, even preceding Aβ deposition (Su et al., 2008). While the reactive oxygen species (ROS) causing this damage likely originate from multiple sources, dysfunctional mitochondria and availability of redox active metals such as Fe++ and Cu+ are thought to play dominant roles (Zhua et al., 2007).

A major consequence of cellular oxidative stress, in the brain and elsewhere, are single or double stranded breaks to the DNA. In response to DNA damage, PARP1 hydrolyzes NAD+ to produce polymers of ADP-ribose (Ying, 2013). We showed previously that NAD+ levels were inversely correlated to measures of oxidative stress in human tissue (Massudi et al., 2012) and in the rat brain (Braidy et al., 2014). Thus, while reduced levels of NAD+ in the brain of living Alzheimer’s sufferers awaits confirmation by non-invasive techniques the consistent findings of oxidative damage in the post mortem brain strongly supports the view that accelerated NAD+ turnover and depletion contribute to the neurological dysfunction in this disease. As interventions targeted at restoring NAD+ have been shown in animal models to support healthy aging and improve metabolic function (Yoshino et al., 2011; Mills et al., 2016) and dementia (Long et al., 2015), strategies to raise NAD+ levels in the human are being actively explored.

The most direct method of increasing NAD+ levels is through intravenous (IV) administration. Though data from experimental research is minimal, the significant clinical benefit of IV NAD+ infusion in alcohol withdrawal has been previously reported (O’Holleran, 1961; Mestayer, 2019). Surprisingly, while the oral administration of NAD+ precursors such as nicotinamide riboside (NR) or nicotinamide mononucleotide (NMN) are being enthusiastically investigated for their impact on NAD+ levels (Yoshino et al., 2011, 2018; Mills et al., 2016; Airhart et al., 2017), the metabolic fate and pharmacokinetic properties of IV NAD+ administration are yet to be reported in humans. This study, therefore, presents for the first time the changes in concentrations of NAD+ and its metabolites during an IV infusion of NAD+ in a cohort of healthy male participants.

## Materials and Methods

### Participants

Eleven (Test *n* = 8, Control *n* = 3) male participants aged 30–55 years were recruited *via* advertisements on radio, TV and social media outlets. All participants had a BMI less than 30 kg/m^2^ (Test average BMI = 27.5 ± 2.5 kg/m^2^; Control average BMI = 24.6 ± 6.5 kg/m^2^), were not diabetic, smoked less than 1 cigarette and consumed less than 2 standard alcoholic drinks per day. Individuals taking lipid lowering or anti-inflammatory drugs, who had a history of liver or renal failure or recently experienced a microbial infection, trauma or any other significant or untreated medical disorders were excluded from the study. Subjects received monetary compensation for their time and participation, regardless of whether they completed the experiment. Participants were not permitted to use natural health products containing NAD+, NR or Nicotinamide within 14 days prior to and during the course of the study.

### Diet Standardization

On the day prior to the NAD+ IV infusion participants consumed an identical niacin-reduced diet and drank only water.

No food was consumed on the day of the study until after the final 8 h blood/urine sample had been collected. Participants were encouraged to drink water to remain normally hydrated. No other beverage types were permitted.

To ensure adequate energy intake was maintained for participants during the 6 h infusion the IV solution also contained 0.1% dextrose, providing approximately 2,000 calories over the 6 h infusion period.

### Infusion Protocol

Participants were randomized to either the Test (*n* = 8) or Control (*n* = 3) group. Participants in the Test group were intravenously administered 750 mg NAD+ in normal saline (Archway Apothecary, Covington, LA, USA) over a 6 h period (infusion rate = ~2 mg/min ≡ 3 μmoles/min). This dosage of NAD+ was derived empirically and reflects a common dosing regimen in clinics (e.g., Springfield Wellness Clinic, Springfield, LA, USA) which regularly provide IV NAD+ infusions in clinical practice. Participants in the Control group were intravenously administered normal saline over a 6 h period.

Clinical administration and supervision of the IV infusions and sample collection were carried out at the Springfield Wellness Center, Springfield, LA, USA.

### Blood Sample Collection

Baseline (TO) blood samples were collected on all participants after an overnight 12 h fast timed to occur immediately prior to the start of the infusion. Additional samples were then collected at 30, 60, 120 (2 h), 360 (6 h) and 480 (8 h) minutes after the start of the infusion.

Whole blood was collected *via* standard venepuncture (opposite arm to infusion site) into a 5 mL non-gel heparinized tube. Immediately after the collection, the blood was centrifuged at 4°C for 10 min at 1,409× *g*.

The plasma and red blood cells fractions were immediately separated and distributed into 5 × 500 μL aliquots each. All aliquots were then immediately frozen and stored at −80°C until analysis.

### Urine Sample Collection

After the collection of a baseline midstream urine sample, participants were asked to void all urine into the receptacle(s) provided at 30 min, 2 h, 6 h and 8 h after initiation of the NAD+ infusion. If participants needed to pass urine intermittently between these time points they were asked to do so into the next successive receptacle. All samples were aliquoted and stored at −80°C immediately upon receipt.

### Analytical Method

Chromatographic separation of NAD+ and related metabolites and MS detection Liquid chromatography coupled to tandem mass spectrometry (LC/MS/MS) was carried out using a Sciex QTRAP 5500 mass spectrometer (Sciex, Redwood City, CA, USA) as previously described (Clement et al., 2018). Briefly, 100 μL of human plasma or urine was extracted in 400 μL of ice-cold methanol, centrifuged at 4°C for 10 min, and filtered through 3 kDa membrane cartridges. Sample extracts were dried under vacuum, reconstituted in 200 μL of 100 mM NH4OAc buffer and transferred into 200 μL glass vials and capped before LC/MS/ MS analysis. Standards and samples (20 μL) were injected onto a Phenomenex NH_2_ column (150 mm· 2 mm· 3 mm) as previously described. A binary solvent gradient consisting of 5 mM NH4OAc pH 9.5 adjusted with ammonia (mobile phase A) and acetonitrile (mobile phase B) with a flow rate of 250 μL/min was used. Initial solvent composition at injection was 25% A, followed by a 2-min gradient to 45% A and a fast gradient ramp to 80% A (0.1 min) that was maintained for 5.9 min, A was increased again to 95% (2 min), held for 13 min, and then reverted to initial conditions (0.1 min) for equilibration, with a total run time of 30 min. The column flow was directed into the MS detector. Calibration curves of individual metabolites were constructed using the peak area ratios (peak area of the metabolite divided by peak area of the selected IS) of each calibrator vs. its concentration.

Internal standards consisted of 2H2NAM (for NAM, methylNAM and ADPR) and ^13^C_5_;-Cyclic AMP (for NMN and NAD+). Note that as isotopic labels are not commercially available for all NAD metabolites, a closely related molecule (structural analog) can also be used (Yamada et al., 2006) provided that it is deemed of similar stability and ionization efficiency during analysis. The internal standards selected in this study have been previously optimized for the related metabolite (Bustamante et al., 2017).

### Safety

The safety of IV NAD+ was assessed using liver function tests and clinical observation of any adverse events. Liver function tests consisted of serum, total bilirubin (bili), alkaline phosphatase (ALP), alanine aminotransferase (ALT), gamma glutamyl transferase (GGT), lactate dehydrogenase (LD) and aspartate aminotransferase (AST).

### Statistical Analysis

Statistical analysis was completed using SPSS version 24 and GraphPad Prism version 8 for windows. An unweighted means, two-way ANOVA with Bonferroni’s multiple comparison *post hoc* test was used to determine if the mean concentration for analysts tested were different over the 8 h period and between Test and Control groups. The Wilcoxon Signed Ranks test was used to determine if differences in mean liver function test concentrations were significant between baseline and 8 h time points. Differences were considered statistically significant when *p* < 0.05.

### Ethics

This study was carried out in accordance with The Code of Ethics of the World Medical Association (Declaration of Helsinki) for experiments involving humans. Ethical approval was obtained from the William Carey University Institutional Review Board, Hattiesburg, MS (Protocol #2017-12). Informed consent was obtained from all participants.

## Results

### Safety

No adverse events were observed during the 6 h infusion with either placebo (saline) or test (NAD+) cohorts.

A significant decrease in activity (1.3, 57, 3.6 units/L) for the liver function enzymes GGT, LD and AST, respectively was observed at 8 h after initiation of the NAD+ infusion (Table 1). No significant change in activity for any liver function marker was apparent at 8 h in the placebo (saline) treated samples, however low sample number may reduce discrimination sensitivity. A significant increase of 2.75 μmoles/L in plasma bilirubin was also observed. However, none of the changes were considered clinically significant.

**Table 1:** The Wilcoxon Signed Ranks test was used to determine if differences in mean liver function test concentrations were significant between baseline and 8 h time points.

	Reference range	Group	Time 0 h Mean (SD)	Time 8 h Mean (SD)	*p* (T0 vs. T8)
Albumin (g/L)	36–47	Test	46.0 (2.5)	45.5 (2.9)	ns
		Control	44.3 (5.6)	42.0 (5.7)	ns
Total protein (g/L)	64–83	Test	72.1 (4.3)	72.0 (4.5)	ns
		Control	76.3 (5.9)	73.7 (4.5)	ns
Total bilirubin (μmol/L)	4–20	Test	9.3 (4.0)	12.0 (3.0)	≤0.05
		Control	13.7 (5.5)	13.3 (4.0)	ns
ALP (units/L)	35–110	Test	77.1 (23.9)	75.9 (21.9)	ns
		Control	67.7 (4.0)	62.7 (2.1)	ns
ALT (units/L)	5–40	Test	40.8 (16.7)	38.9 (15.1)	ns
		Control	18.7 (4.9)	17.7 (4.0)	ns
GGT (units/L)	5–50	Test	25.1 (17.4)	23.8 (16.9)	≤0.05
		Control	23.3 (7.6)	21.7 (7.2)	ns
LD (units/L)	20–250	Test	256.8 (66.3)	198.8 (78.0)	≤0.05
		Control	133.3 (12.7)	146.3 (43.5)	ns
AST (units/L)	10–40	Test	29.6 (6.7)	26.0 (6.7)	≤0.05
		Control	22.0 (6.1)	21.7 (9.0)	ns

*Differences were considered statistically significant when *p* < 0.05*.

### Plasma

A continuous infusion of NAD+ at a rate of 3 μmoles/min resulted in a significant (398%) increase in plasma NAD+ levels only at the 6 h time point (i.e., end of infusion) relative to baseline (*p* < 0.0001). This was significantly different from the 6 h saline-treated control (*p* < 0.001).

NAD+ levels remained elevated at 8 h (i.e., 2 h post-infusion) relative to baseline and saline treated control samples.

**Figure 1:**
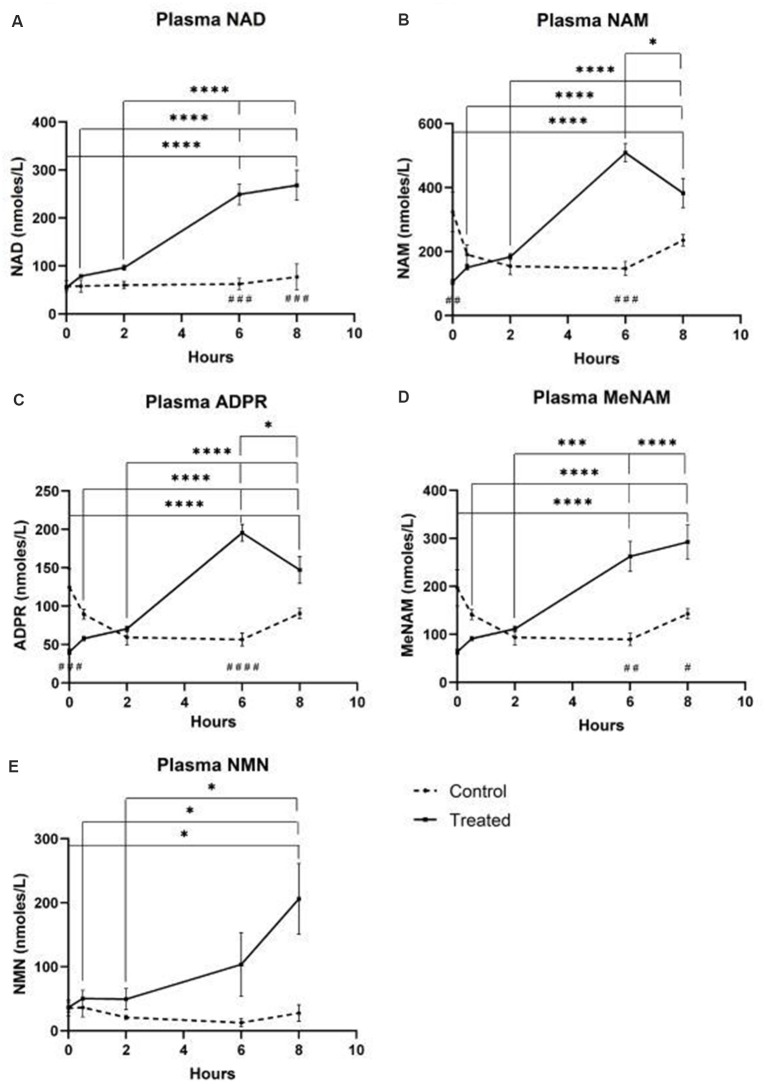
Changes in plasma nicotinamide adenine dinucleotide (NAD+) and metabolites over 8 h [1st 6 h consisting of a continuous (3 μmoles/min) NAD+ IV infusion]. **(A)** NAD+, **(B)** Nicotinamide (NAM), **(C)** adenosine phosphoribose (ADPR), **(D)** methyl nicotinamide (meNAM), **(E)** nicotinamide mononucleotide (NMN). *p < 0.05, **p < 0.01, ***p < 0.001, ****p < 0.0001, as indicated. Two-way ANOVA with Bonferroni’s multiple comparison *post hoc* test was used to determine if the mean concentration for analysts tested were different over the 8 h period and between Test (*n* = 8) and Control (*n* = 3) groups.

Plasma NAD+ levels did not change significantly from baseline across the 8 h assessment period in saline treated control samples (Figure 1A).

Similar to the changes observed for NAD+, plasma levels of the NAD+ metabolite nicotinamide (NAM) increased significantly by 409% at the end of the NAD+ infusion (i.e., 6 h) relative to baseline (*p* < 0.0001). This was likewise significantly different to the 6 h saline treated control (*p* < 0.001).

At the 8 h time point (i.e., 2 h after end of infusion), NAM levels between the control and treated groups were no longer significantly different (*p* > 0.05).

NAM levels for saline treated control samples did not change significantly across the 8 h time period (*p* > 0.05, Figure 1B).

Consistent with the changes observed for NAM, plasma levels of the NAD+ metabolite ADPR increased significantly by 393% at the end of the NAD+ infusion (i.e., 6 h) relative to baseline (*p* < 0.0001). This was significantly different to the 6 h saline treated control (*p* < 0.0001).

At the 8 h time point (i.e., 2 h after the end of infusion), ADPR levels remained 305% above baseline (*p* < 0.0001). However, this was not significantly greater than ADPR levels in the saline treated control samples at the same time point (*p* > 0.05).

ADPR levels for saline treated control samples did not change significantly across the 8 h time period (*p* > 0.05, Figure 1C).

A Spearman’s correlation analysis between group averages across the 8 h time points for the two NAD+ catabolic metabolites, NAM and ADPR, produced a correlation coefficient of 1.00 (*p* < 0.001).

Again, consistent with the changes observed for NAM, plasma levels of the NAM metabolite, methyl-nicotinamide (meNAM) increased significantly to 350% at the end of the NAD+ infusion (i.e., 6 h) relative to both baseline (*p* < 0.0001) and the 6 h saline treated control (*p* < 0.01).

At the 8 h time point (i.e., 2 h after end of infusion) meNAM levels remained 393% above baseline (*p* < 0.0001) and significantly greater than the saline treated control samples at the same time point (*p* < 0.05).

meNAM levels for saline treated control samples did not change significantly across the 8 h time period (*p* > 0.05, Figure 1D).

Plasma levels of NMN, a metabolite of NAM *via* the anabolic salvage pathway, was significantly elevated (472%) only at the 8 h time point (i.e., 2 h after end of infusion, *p* < 0.05).

NMN levels for saline treated control samples did not change significantly across the 8 h time period (*p* > 0.05, Figure 1E).

### Urine

The continuous IV infusion of NAD+ at a rate of 3 μmoles/min resulted in a significant (538%) increase in the urine NAD+ excretion rate at the 6 h time point (i.e., end of infusion) relative to that excreted at 30 min (*p* < 0.001). This was also significantly different to the amount of urine NAD+ excreted at 6 h by the saline treated controls (*p* < 0.05).

The amount of NAD+ excreted in urine decreased by 43% (*p* < 0.05) at 8 h (i.e., 2 h post-infusion) relative to the peak excretion at the 6 h time point.

**Figure 2:**
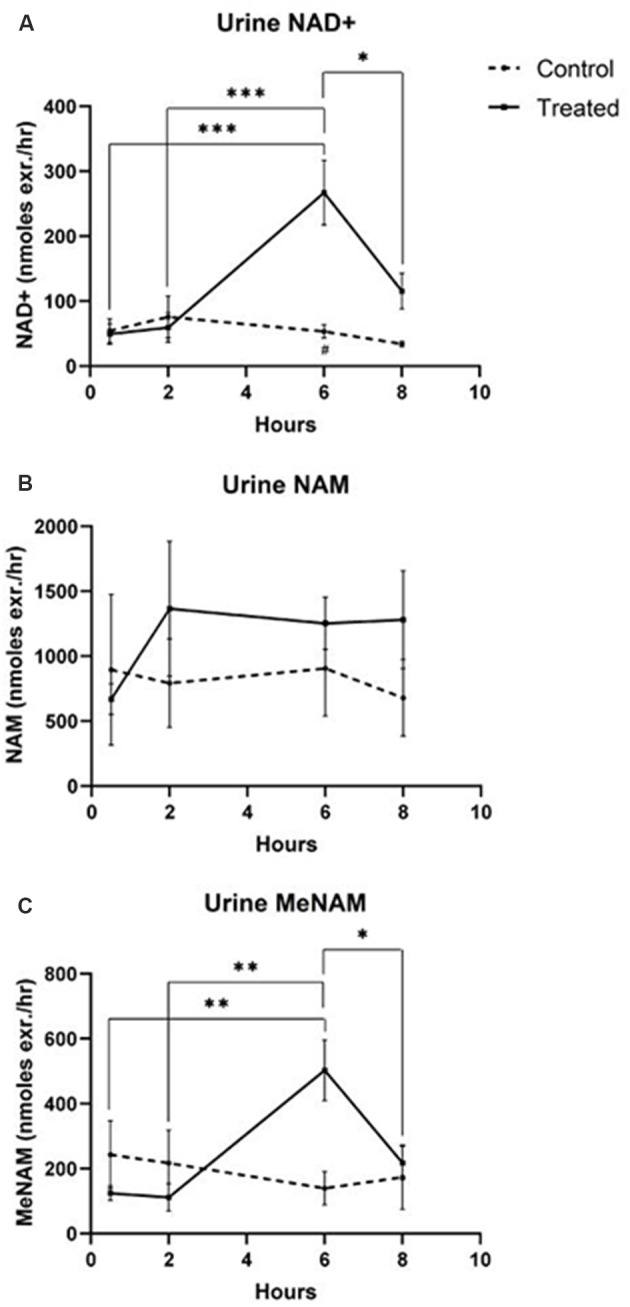
Changes in urine NAD+ and metabolites over 8 h (1st 6 h consisting of a continuous, 3 μmoles/min, NAD+ IV infusion). **(A)** NAD+, **(B)** NAM, **(C)** meNAM. *p < 0.05, **p < 0.01, ***p < 0.001, ****p < 0.0001, as indicated. Two-way ANOVA with Bonferroni’s multiple comparison *post hoc* test was used to determine if the mean concentration for analysts tested were different over the 8 h period and between Test (*n* = 8) and Control (*n* = 3) groups.

The urine excretion rate for NAD+ did not change significantly across the 8 h assessment period in saline treated control samples (Figure 2A).

The excretion rate of the NAD+ metabolite NAM did not change significantly across the 8 h test period and was not different to the observed NAM excretion rate for the saline treated controls (Figure 2B).

The urine excretion rate of the NAM metabolite meNAM was significantly increased (403%) at the 6 h time point (i.e., end of infusion) relative to that observed at 30 min (*p* < 0.01). The amount of meNAM excreted in urine decreased by 43% (*p* < 0.05) at 8 h (i.e., 2 h post-infusion) relative to the peak excretion at the 6 h time point.

The urine excretion rate for meNAM did not change significantly across the 8 h assessment period in saline treated controls (Figure 2C).

## Discussion

A growing interest in NAD+ based therapies including NAD+ infusions has highlighted the need for a clearer understanding of the fate of NAD+ and its metabolites following IV administration. The current study documents changes in levels of NAD+ and key metabolites in both plasma and urine over 8 h using a typical clinical dosing regimen of 750 mg NAD+ administered IV over a 6 h period.

Importantly infusion of NAD+ did not produce any observable adverse events in the test cohort but rather reduced plasma activities of enzymes indicative of hepatic stress such as the intrahepatic LD and AST and the post-hepatic (bile duct) enzyme GGT suggesting that the integrity of both intra hepatic and post-hepatic tissue was enhanced even within the relatively short 8 h time frame. The rise in bilirubin, a red cell degradation product, at 8 h may reflect either a very small increase in red cell turnover, as can occur due to infusion induced hemeolysis, or reduced heme metabolism (Table 1). However, given the very low magnitude of this change, this was not considered clinically relevant.

As expected, in saline-treated (i.e., control) participants, plasma levels of NAD+ and metabolites NAM and ADPR and the NAM metabolites, NMN and meNAM remained essentially unchanged across the 8 h period. However, an apparent decrease in NAM, meNAM and ADPR were observed between 30 min and 6 h, most likely due to a saline dilution effect. Consistent with this notion, values for these analytes were seen to return to baseline levels at 8 h (i.e., 2 h after the end of the saline infusion).

Unexpectedly however in NAD+ infused participants, plasma NAD+ levels failed to rise until after the 2 h time point reaching a maximum of ~400% above baseline for NAD+ and metabolites NAM, meNAM and ADPR (398%, 409%, 393%, respectively) only at the 6 h time point (Figures 1A–E). This was internally consistent with the peak of urinary excretion for both NAD+ and meNAM also occurring at 6 h before rapidly decreasing after the end of the infusion (Figures 2A–C).

NAD+ was infused at a constant rate of 3 μmoles/min. Therefore 90 μmoles of NAD+ was being infused directly into the vascular compartment every 30 min delivering a total of 1,080 μmoles by the end of the infusion at 6 h. Intravascular mixing from any point of infusion occurs within ~2 min; assuming an average blood volume of 5, the added NAD+, in the absence of significant metabolism or absorption, would correspond to an additional rise in (NAD+) of at least 18 μM, every 30 min across the 6 h of infusion. While an increase of this magnitude is well within this study’s analytical detection limits no increase in either NAD+ or its metabolites, were observed until after 2 h (i.e., at the 6 h time point) in either plasma or urine. This unexpected observation indicates a rapid, and for at least the first 2 h, complete tissue uptake and/or metabolism of NAD+ and/or its metabolites.

**Figure 3:**
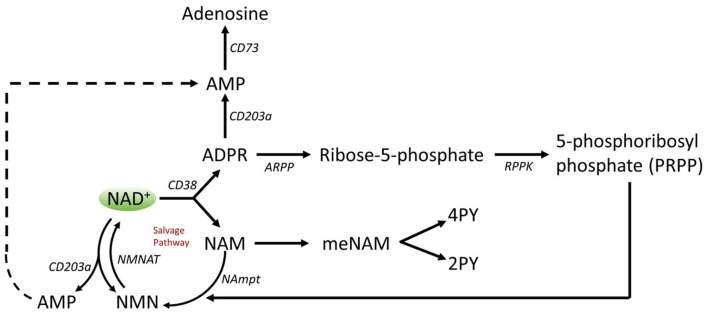
Potential extracellular catabolism of exogenously supplied NAD+ through activity of ectoenzymes CD38 (ADP-ribose (ADPR) synthase), CD203a (NAD+ pyrophosphatase), CD 73 (5′-nucleotidase), CD157-ADP-ribosyl cyclase 2. Abbreviations: NAM, nicotinamide; NMN, nicotinamide mononucleotide; ADPR, adenosine diphosphate riboside; meNAM, methyl nicotinamide; 4PY, N-methyl-4-pyridone-3-carboxamide; 2PY, methyl-2-pyridone-5-carboxamide; ARPP, ADP ribose pyrophosphatase; RPPK, ribosyl pyrophosphokinase; NAmpt, nicotinamide phosphoribosyltransferrase; NMNAT, nicotinamide mononucleotideadenyltransferase; CX-43, connexin 43.

A number of enzymes can achieve effective NAD+ catabolism including the sirtuins (SIRTs 1–7) the adenosine diphosphate (ADP)–ribose transferases (ARTs) and poly(ADP-ribose) polymerases (PARPs 1–17) and the cyclic ADP-ribose (cADPR) synthases (CD38, CD157). Extracellular NAD+ pyrophosphatases present in human serum can also degrade NAD+ to AMP and NMN (Schmidt-Brauns et al., 2001). The cell-surface protein CD73 also converts NMN into NR, which easily crosses cell membranes for potential resynthesis to NAD+. Importantly the NAD+ catabolizing glycohydrolases, CD38 and CD157, ecto-nucleotide pyrophosphatase (CD203a) and the NMN catabolizer CD73 are ectoenzymes found on a wide variety of cells including lymphoid, granulocytic, neuronal and endothelial cells (Wei et al., 2014) and plasma soluble NAD glycohydrolases may also be present (Figure 3; Funaro et al., 2009). The parallel rise of plasma NAM and ADPR (correlation coefficient of 1.000, *p* < 0.001, data not shown) strongly suggests that at least by 6 h a major fate of NAD+ is metabolism through cleavage of the glycosidic ADPribose–nicotinamide linkage to NAM and ADPR, by-products symptomatic of NAD glycohydrolase (e.g., CD38) activity. This is consistent with evidence by others where CD38, in particular, has been shown to have a primary role in controlling extracellular NAD+ levels (Wei et al., 2014). Adult human erythrocytes are CD38 positive and express high levels of NAD+ glycohydrolase activity, cleaving exogenous NAD+ to supply erythrocytes with ADP ribose that can be efficiently taken up into the cell (Kim et al., 1993; Albeniz et al., 2004). The absence of any rise in either NAD+ or metabolites, in plasma or urine, until after the first 2 h of the infusion indicates that NAD+ and/or its metabolites are trafficked out of the extracellular vascular space and efficiently sequestered into tissue or extravascular compartments as NAD+ and/or its metabolites during this time period.

**Figure 4:**
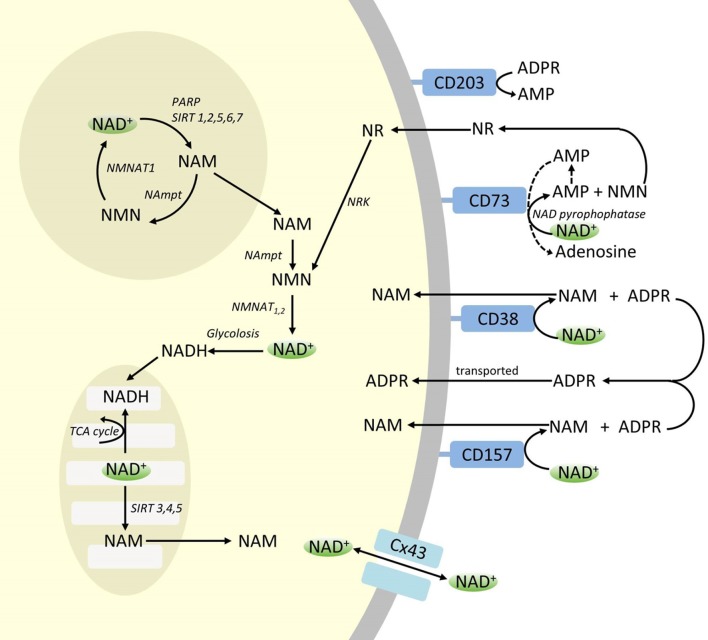
Potenital Intra and extracellular fate of exogenously supplied NAD+. Abbreviations: CD38 [cyclic ADP-ribose (cADPR) synthase], CD203a (NAD+ pyrophosphatase), CD 73 (5′-nucleotidase). NAM, nicotinamide; NMN, nicotinamide mononucleotide; ADPR, adenosine diphosphate riboside; meNAM, methyl nicotinamide; 4PY, N-methyl-4-pyridone-3-carboxamide; 2PY, methyl-2-pyridone-5-carboxamide; ARPP, ADP ribose pyrophosphatase; RPPK, ribosyl pyrophosphokinase; NAmpt, nicotinamide phosphoribosyltransferrase; NMNAT, nicotinamide mononucleotideadenyltransferase; PARP [poly ADPribose polymerase; SIRT [sirtuin]; NRK [nicotinamide riboside kinase].

Though PARP and sirtuins are involved in NAD+ catabolism, as intracellular enzymes with representatives in cytoplasm, nucleus and mitochondria they are unlikely to directly impact extracellular NAD+ levels. However, these enzymes may be expected to respond to changes in intracellular concentrations of NAD+, NAM and NR that may arise from exogenously supplied NAD+. Figure 4 summarizes schematically the possible fates of exogenous NAD+.

While significant capacity exists for the rapid degradation of the exogenously supplied IV NAD+ into constituent metabolites, it is also worth noting that uptake of extracellular NAD+ may also be occurring. As NAD+, has an overall negative charge it is unable to cross cellular membranes passively and therefore must be actively transported across the membrane. That this occurs has been shown by a number of researchers who have reported that exogenous NAD+ applied to a variety of human cell types does indeed result in a significant elevation of intracellular NAD+ (Ying et al., 2003; Zhu et al., 2005; Billington et al., 2008; Pittelli et al., 2011; Felici et al., 2013). While the mechanism(s) involved are not yet fully characterized, Alano et al. (2010) reported that exogenous NAD+ could enter neurons through P2X7 gated channels and others have consistently observed the transport of NAD+ across membranes by connexin 43 (CX43) hemichannels, even at concentrations as low as 250 pM (Billington et al., 2008). As connexins have a wide distribution in human tissue and CX43 appears to be the most ubiquitous connexin in many cell types the potential for rapid uptake of NAD+ cannot be discounted.

Thus cellular uptake and/or metabolism of NAD+ and metabolites NAM and ADPR and secondary metabolites meNAM and NMN appeared to proceed at pace with the 3 μmoles/min NAD+ infusion for at least the first 2 h. Either NAD+ and/or the primary metabolites tested are being efficiently sequestered during this first 2–6 h period or secondary metabolites are also being formed. ADPR can be recycled to produce NAD+ from NAM (Figure 4) or further metabolized *via* ectoenzymes such as CD203a (NAD+ pyrophosphatase) to produce AMP which can be further rapidly metabolized to adenosine by CD73 a 5′-nucleotidase (Bogan and Brenner, 2010; Horenstein et al., 2016; Morandi et al., 2018). In support of this hypothesis, an increase in blood adenosine levels has been recognized by others as a consequence of extracellular NAD+ infusion (Szczepañska-Konkel et al., 2003). NAM can also be metabolized to NMN *via* the salvage pathway before resynthesis to NAD+ (Figures 3, 4) or acted on by hepatic methyltransferases to produce N1-methylnicotinamide which may either be excreted directly or further converted to N-methyl-2-pyridone-5-carboxamide (2PY, +99% of meNAM) and N-methyl-4-pyridone-3-carboxamide (4PY, ~0.25% of meNAM) before excretion (Shibata and Matsuo, 1989; Okamoto et al., 2003).

It is evident that at the dosing regimen used the mechanisms involved in the metabolism and sequestering of NAD+ and metabolites have reached saturation sometime after 2 h resulting in a significant elevation of plasma NAD+ and accumulation of all metabolites tested (NMA, ADPR, meNAM and NMN) at 6 h. As mentioned previously these data support the view that one major pathway for NAD+ metabolism under these conditions is the cleavage of the glycosidic bond, by ectoenzymes such as CD38 to produce NAM and ADPR. However, the rise in plasma NMN after 2 h also suggests that exogenous NAD+ is likely acted on by extracellular NAD+ pyrophosphatases, elevating plasma NMN and releasing AMP as an additional metabolite.

In summary, this study revealed for the first time that: (a) at a flow rate of 3 μmole/min all exogenously infused NAD+ was rapidly and completely removed from the plasma for at least the first 2 h; (b) the increase in metabolic bi-products analyzed is consistent with NAD+ glycohydrolases and NAD+ pyrophosphatase activity; and (c) the urinary excretion products arising from NAD+ infusion include native NAD+ and meNAM but not NAM.

While the findings of this investigation are novel and go some way to advancing our understanding of the timed fate of exogenous NAD+ in humans, limitations were identified. To improve overall metabolic reckoning future studies should investigate changes in red cell NAD+ and metabolites and urinary excretion of the secondary meNAM metabolites, 2PY and 4PY. In addition, it will likely be useful to assess the impact of exogenous NAD+ on changes in purine metabolism which should include at least assessment of plasma and red cell AMP and adenosine. The use of animal models may also help to clarify the relative involvement of the various metabolic pathways through the use of suitable pharmacological inhibitors and tissue sampling.

In conclusion, this study was able to reveal for the first time some very useful, previously unknown, information about the fate of exogenous IV NAD+ in humans including, the overall safety and tolerability of an IV NAD+ infusion at a rate of 3 μmoles/min, the rapid sequestering of NAD+ from the plasma, the likely contribution of both NAD+ glycohydrolase and NAD+ pyrophosphate activity in the metabolism of NAD+ and the apparent efficient renal tubular reabsorption of NAM. However additional research is required to fully reveal the complex metabolic fate of this important molecule. Further, characterization of these changes will help progress the development and improvement of NAD+ based treatment regimens for disorders which are likely to benefit from increased NAD+ availability including conditions requiring increased cellular regeneration and repair such as Alzheimer’s and other neurodegenerative dementias.

## Data Availability

The datasets generated for this study are available on request to the corresponding author.

## Ethics Statement

The studies involving human participants were reviewed and approved by William Carey University Institutional Review Board, Hattiesburg, MS (Protocol #2017-12). The patients/participants provided their written informed consent to participate in this study.

## Author Contributions

RG participated in study design and oversight, critical discussion and manuscript authorship. JBer participated in study design, critical discussion, data collection, statistical analysis, manuscript drafting and manuscript review. RM participated in study design, critical discussion, clinical supervision and manuscript review. NB participated in biochemical analysis and manuscript review. JW participated in study design, critical discussion and manuscript review. JBen participated in data collection, and manuscript review. SB participated in study design and manuscript review. All authors have reviewed, read and approved the final version of the manuscript, and agree with the order of presentation of the authors.

## Conflict of Interest Statement

RM is a director of NAD+ Research Inc., and medical director of the Springfield Wellness Center which uses IV NAD as a clinical therapy. SB received consulting fees from NAD+ Research Inc. The remaining authors declare that the research was conducted in the absence of any commercial or financial relationships that could be construed as a potential conflict of interest.
